# Lung volume reduction surgery *versus* endobronchial valves: a randomised controlled trial

**DOI:** 10.1183/13993003.02063-2022

**Published:** 2023-04-27

**Authors:** Sara C. Buttery, Winston Banya, Rocco Bilancia, Elizabeth Boyd, Julie Buckley, Neil J. Greening, Kay Housley, Simon Jordan, Samuel V. Kemp, Alan J.B. Kirk, Lorna Latimer, Kelvin Lau, Rod Lawson, Adam Lewis, John Moxham, Sridhar Rathinam, Michael C. Steiner, Sara Tenconi, David Waller, Pallav L. Shah, Nicholas S. Hopkinson

**Affiliations:** 1 National Heart and Lung Institute, Imperial College London, London, UK; 2 Royal Brompton and Harefield Hospitals, London, UK; 3 Department of Thoracic Surgery, West of Scotland Regional Heart and Lung Centre, Golden Jubilee National Hospital, Glasgow, UK; 4 Department of Respiratory Sciences, University of Leicester, Leicester, UK; 5 Institute for Lung Health, National Institute for Health Research Leicester Biomedical Research Centre – Respiratory, Glenfield Hospital, Leicester, UK; 6 Northern General Hospital, Sheffield, UK; 7 St Bartholomew's Hospital, London, UK; 8 Department of Health Sciences, Brunel University London, Uxbridge, UK; 9 King's College, London, UK

## Abstract

**Background:**

Lung volume reduction surgery (LVRS) and bronchoscopic lung volume reduction (BLVR) with endobronchial valves can improve outcomes in appropriately selected patients with emphysema. However, no direct comparison data exist to inform clinical decision making in people who appear suitable for both procedures. Our aim was to investigate whether LVRS produces superior health outcomes when compared with BLVR at 12 months.

**Methods:**

This multicentre, single-blind, parallel-group trial randomised patients from five UK hospitals, who were suitable for a targeted lung volume reduction procedure, to either LVRS or BLVR and compared outcomes at 1 year using the i-BODE score. This composite disease severity measure includes body mass index, airflow obstruction, dyspnoea and exercise capacity (incremental shuttle walk test). The researchers responsible for collecting outcomes were masked to treatment allocation. All outcomes were assessed in the intention-to-treat population.

**Results:**

88 participants (48% female, mean±sd age 64.6±7.7 years, forced expiratory volume in 1 s percent predicted 31.0±7.9%) were recruited at five specialist centres across the UK and randomised to either LVRS (n=41) or BLVR (n=47). At 12 months follow-up, the complete i-BODE was available in 49 participants (21 LVRS/28 BLVR). Neither improvement in the i-BODE score (LVRS −1.10±1.44 *versus* BLVR −0.82±1.61; p=0.54) nor in its individual components differed between groups. Both treatments produced similar improvements in gas trapping (residual volume percent predicted: LVRS −36.1% (95% CI −54.6– −10%) *versus* BLVR −30.1% (95% CI −53.7– −9%); p=0.81). There was one death in each treatment arm.

**Conclusion:**

Our findings do not support the hypothesis that LVRS is a substantially superior treatment to BLVR in individuals who are suitable for both treatments.

## Abstract 2

**In this first randomised study to compare lung volume reduction surgery and endobronchial valve placement in people who are suitable for both treatments, surgery did not produce substantially superior outcomes at 1** **year post-procedure** http://bit.ly/3D0DjoN

## Introduction

COPD is a common and often disabling condition which is now the third largest cause of death worldwide [1]. Breathlessness, exercise limitation and mortality in COPD are all associated with increased lung volumes occurring due to airflow obstruction and increased lung compliance. COPD is progressive, and despite optimum care including smoking cessation, pharmacotherapy and pulmonary rehabilitation, many patients remain breathless and limited in everyday activities [2, 3]. Surgical and bronchoscopic approaches to lung volume reduction are available which can bring substantial benefits in appropriately selected individuals, although both are also associated with some risk [4]. Lung volume reduction surgery (LVRS) involves removing the worst affected area of emphysematous lung, allowing the remaining healthier and less compliant lung to function more effectively, with the respiratory muscles working at less of a mechanical disadvantage [5]. LVRS has been shown to improve survival, exercise capacity and quality of life in appropriately selected patients with heterogeneous emphysema and poor exercise capacity [5–9], and is recommended in national and international guidelines for the management of COPD [10, 11]. However, uptake has been limited, due in part to exaggerated concerns about surgical morbidity and mortality [9, 12]. In modern clinical practice, morbidity and mortality from the procedure are substantially lower [9, 13] than was the case in trials conducted around the turn of the century [5]. Common complications that may be associated with LVRS include prolonged air leak, infection and need for revision of procedure.

An alternative lung volume reduction approach is endobronchial placement of valves to the airways supplying the most emphysematous lobe causing it to deflate. This form of bronchoscopic lung volume reduction (BLVR) can produce lobar atelectasis and is intended to achieve similar benefits to LVRS but with less morbidity [14–18]. It is only effective in the absence of interlobar collateral ventilation [17, 19]. If this is present, air can enter the target lobe from an adjacent lobe and atelectasis does not occur. In patients with a heterogeneous pattern of emphysema and no collateral ventilation, valve placement produces significant improvements in lung function, exercise capacity and health status [4, 14, 15, 17]. There is also evidence to suggest that endobronchial valves may benefit those with a homogenous pattern of emphysema [16]. The most important complication post-BLVR is pneumothorax, which occurs in up to 30% of cases [20] and can on occasion be fatal. Acute exacerbation-like events are also common, while valve expectoration or misplacement can necessitate additional procedures [21, 22].

People with heterogeneous emphysema and an absence of collateral ventilation may therefore benefit from either BLVR or LVRS, but there are no direct comparison data on the relative value of the two procedures to guide clinical decision making. The aim of our study was to determine whether LVRS produces a health benefit at 1 year that is sufficiently greater [23] than BLVR to be likely to influence choice of procedure.

## Methods

### Study design and participants

The CELEB study was a multicentre, randomised controlled, parallel-group superiority trial in which patients with COPD who were considered by a lung volume reduction multidisciplinary team (MDT) meeting to be suitable candidates for both forms of targeted lung reduction therapy, and who did not have collateral ventilation on Chartis assessment (PulmonX, Redwood City, CA, USA), were randomised to either BLVR or unilateral LVRS (supplementary figure S1).

Ethical approval was obtained from Fulham Research Ethics Committee (London, UK) (REC reference: 16/LO/0286). The trial protocol has been published previously [24]. The trial was registered prospectively at the ISRCTN registry with identifier ISRCTN19684749. A trial steering committee with an independent chair met quarterly to review progress, conduct, safety and consistency of trial processes and decision making at each centre throughout the course of the trial.

Participants were recruited at five UK hospital sites which had an established MDT meeting dedicated to identifying suitable candidates for lung volume reduction: Royal Brompton Hospital (London), Glenfield Hospital (Leicester), St Bartholomew's Hospital (London), Northern General Hospital (Sheffield) and Golden Jubilee National Hospital (Glasgow).

Eligibility criteria: significant airflow obstruction (forced expiratory volume in 1 s (FEV_1_) <60% predicted), hyperinflation (total lung capacity >100% predicted, residual volume (RV) >170% predicted) [25], considered to have heterogeneous emphysema based on computed tomography (CT) and lung perfusion, and with an absence of collateral ventilation (>90% interlobar fissures on CT and negative Chartis assessment).

Exclusion criteria: smoked in previous 3 months [25], pulmonary fibrosis or other major comorbidity that could affect survival or mean that lung volume reduction procedures were unlikely to be effective and hypoxaemia arterial oxygen tension <7.0 kPa. For complete inclusion/exclusion criteria, see supplementary appendix 1.

All participants were assumed to be medically optimised and required to have undergone a course of pulmonary rehabilitation within the 12 months preceding trial enrolment. The clinical MDT then decided on whether a patient was suitable for both interventions and if there was equipoise between the two options. It was only after this point in the normal clinical process that a trial screening visit was arranged and written informed consent was obtained.

### Randomisation and masking

Randomisation to the treatment arm occurred only after the MDT meeting and once participants had undergone a fibreoptic bronchoscopy to allow for assessment of the presence of collateral ventilation using the Chartis system. People who were collateral ventilation-positive exited the study as valves would not be effective, so there was no longer equipoise (supplementary figure S1). Randomisation was completed by a trial coordinator at each individual centre, on a 1:1 basis using a computer-generated random sequence from Sealed Envelope (www.sealedenvelope.com) that used centre and i-BODE score (>7/≤7 points) for stratification. Blinding of trial participants and the trial coordinator was not possible due to the nature of the interventions, but primary outcome data were collected by an assessor with no knowledge of participant procedure and participants were asked to not reveal their treatment allocation.

### Procedures

LVRS, to remove the most emphysematous part of the target lung, was carried out by a thoracic surgeon under general anaesthesia, primarily using either unilateral video-assisted thoracoscopic surgery or unilateral robot-assisted surgery. Where required, an open thoracotomy was performed at the discretion of the surgeon. As per usual clinical practice, participants initially went to the high dependency unit post-operatively and were transferred to ward-based care as soon as deemed medically stable, for further post-operative management, prior to discharge.

BLVR, placing Zephyr endobronchial valves (PulmonX) to occlude the target lobe, was performed *via* bronchoscopy by an operator experienced in placing endobronchial valves, either under conscious sedation or general anaesthesia, as necessary. A chest radiograph was performed 1 h after the procedure and participants were required to spend a minimum of 3 nights post-procedure as an inpatient in case a pneumothorax was to occur. Further details about procedures have been published previously [24] and a summary can be accessed in supplementary appendix 2. Participants were followed up at 3 and 12 months post-procedure. Trial outcomes were assessed and recorded at baseline, and at 3 and 12 months after intervention (supplementary figure S1).

### Outcomes

The primary outcome for the trial was the between-group difference in i-BODE score from baseline to 12 months post-procedure. This composite measure of disease severity is made up of body mass index (BMI), airflow obstruction (FEV_1_ % pred), Medical Research Council (MRC) dyspnoea score and exercise capacity (incremental shuttle walk test (ISWT)), and has been related to prognosis in a number of settings [26]. A score is assigned based on these four criteria, with the highest possible score being 10 and lowest being 0. Higher scores are associated with increased mortality. Secondary outcomes were: health-related quality of life (COPD Assessment Test (CAT) score), patient experience of physical activity (MoveMonitor; McRoberts, The Hague, The Netherlands) assessed using the clinic visit PROactive Physical Activity in COPD (c-PPAC) score which has domains of Amount and Difficulty [27], change in RV (% pred), and change in fat-free mass index (FFMI). Procedure-related morbidity data were also compared, including length of hospital stay, days with intercostal drainage, days spent in intensive care and need for further intervention, including pneumothorax and other complications.

### Statistical analysis

Sample size calculation was based on a study comparing change in BODE score 3 months post-LVRS between survivors and non-survivors at 5 years [23]. We took the difference between these groups (1.5 points) to be sufficiently important to influence clinical decision making. For practical purposes we have taken the BODE and the i-BODE score to be equivalent [26]. Based on a standard deviation of 1.8 for change in i-BODE score and taking a 5% significance level and 90% statistical power, we would require 34 participants in each arm, and allowing for a 10% dropout rate, a recruitment target of 76 participants.

Changes in outcome measures between groups were analysed using independent t-tests where normally distributed, otherwise the Wilcoxon rank-sum (Mann–Whitney) test was used. Treatment effect was reported as difference between means with associated 95% confidence intervals or the Hodges–Lehman estimate with its associated 95% confidence intervals. All analyses were performed according to a predefined statistical analysis plan [24], based on an intention-to-treat [28] principle. A sensitivity analysis, where missing data were imputed under a missing at random assumption, was performed, imputing data on all variables with missing data. These values were replaced using multiple imputation by chained equations, including the 3-month i-BODE score components. For each variable 10 imputed datasets were created and Rubin's rule was used to obtain an overall estimate [29]. Data were analysed by a healthcare statistician (W.B.) using Stata version 6.1 (StataCorp, College Station, TX, USA).

## Results

**FIGURE 1:**
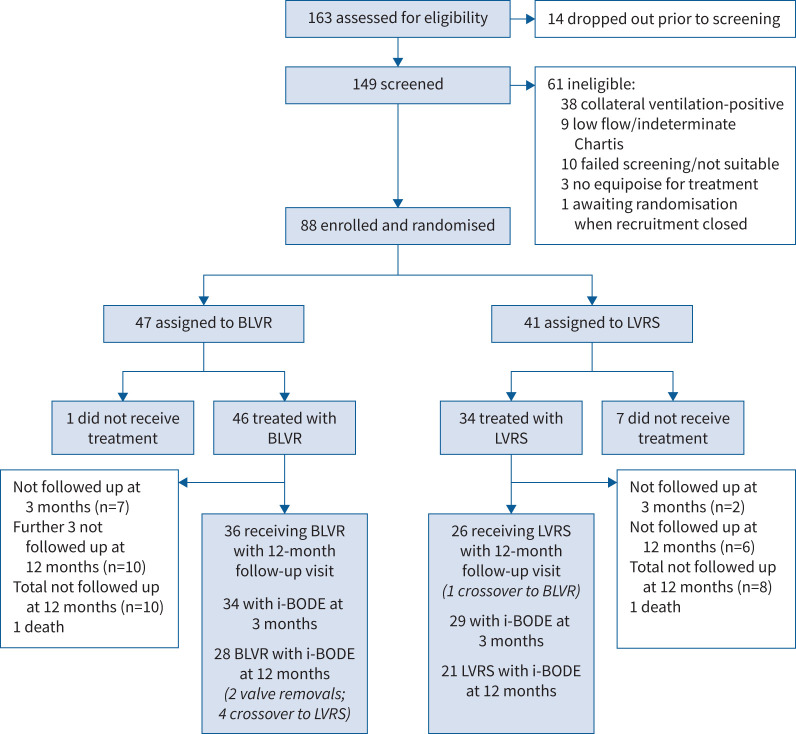
Trial profile. BLVR: bronchoscopic lung volume reduction; LVRS: lung volume reduction surgery.

Between 16 September 2016 and 22 July 2019, 163 patients were assessed for their eligibility to be enrolled in the trial. Of 149 patients who were screened and thought on the basis of their CT scan to be collateral ventilation-negative, 38 (26%) were collateral ventilation-positive on Chartis assessment and nine (6%) had a low flow or indeterminate Chartis. 88 eligible participants were randomly assigned to either LVRS (n=41) or BLVR (n=47) (figure 1). Of the randomised participants, 46 (52%) were male, mean±sd age 64.6±7.7 years, FEV_1_ % pred 31.0±7.9%, RV % pred 240.1±39.0%, 48±27 pack-years smoking history and median (range) exacerbation rate 2 (1–3) per year. 87 (98.9%) described their ethnicity as White and one (1.1%) as Middle Eastern. Groups were well matched in terms of lung function parameters, exercise capacity, health-related quality of life and i-BODE score (table 1). Among participants who were initially thought to be eligible for the study at the MDT meeting but later excluded after full screening, the most common reason for exclusion (n=47 (77.0%)) was the presence of collateral ventilation at Chartis assessment or an indeterminate collateral ventilation measurement (figure 1).

**TABLE 1:** Baseline characteristics of whole cohort and by treatment allocation

	**All (n=88)**	**LVRS (n=41)**	**BLVR (n=47)**
**Age (years)**	64.6±7.7	65.2±7.9	64.0±7.6
**Gender**			
Female	42 (47.7)	22 (53.7)	20 (42.6)
Male	44 (52.3)	19 (46.3)	27 (57.4)
**Ethnicity**			
White	87 (98.9)	40 (97.6)	47 (100.0)
Middle Eastern	1 (1.1)	1 (2.4)	0 (0.0)
**Exacerbations^#^**	2 (1–3)	1.5 (1–2.5)	3 (1–4)
**Emergency department attendances^#^**	0 (0–1)	0 (0–0)	0 (0–1)
**Hospital admissions^#^**	0 (0–0)	0 (0–0)	0 (0–1)
**Hospital days^#^**	0 (0–0)	0 (0–0)	0 (0–1)
**LTOT use**	1 (1.1)	0 (0.0)	1 (2.1)
**Ambulatory oxygen use**	8 (9.1)	5 (12.2)	4 (8.5)
**i-BODE score**	5.9±1.5	5.9±1.4	5.9±1.6
**BMI (kg·m^−2)^**	23.7±3.7	23.8±3.9	23.6±3.5
**FEV_1_ (% pred)**	31.0±7.9	32.0±7.7	30.2±8.0
**MRC dyspnoea score**	4 (3–4)	4 (4–4)	4 (3–4)
**ISWT distance (m)**	210 (125–265)	200 (130–260)	210 (120–270)
**Other lung function parameters**			
FEV_1_ (L)	0.80±0.22	0.81±0.21	0.80±0.22
FVC (L)	2.82±0.81	2.80±0.70	2.84±0.90
FVC (% pred)	86.1±19.0	88.2±20.0	84.3±18.1
FEV_1_/FVC ratio	28.03 (24.08–35)	28.02 (25.5–32)	29.0 (23.89–35.4)
TLC (L)	8.08±1.83	7.91±1.87	8.20±1.81
TLC (% pred)	142.0±14.1	142.4±13.8	141.7±14.5
RV (L)	5.3±1.2	5.2±1.3	5.4±1.1
RV (% pred)	240.1±39.0	236.9±39.3	242.9±39.0
RV/TLC ratio	64.0±30	63.9±5.6	64.0±7.0
FRC (L)	6.3±1.4	6.1±1.5	6.4±1.3
FRC (% pred)	195.5 (182–211.5)	197.5 (182.4–209.1)	194.5 (178.9–214.6)
*T*_LCO_ (% pred)	35.8±10.0	35.9±10.6	35.7±9.5
*K*_CO_ (% pred)	46.7±13.9	46.4±14.4	47.1±13.5
**Other secondary outcomes**			
FFMI (kg·m^−2^)	30.9±5.7	30.9±6.2	30.9±5.3
CAT score	23.1±6.4	23.9±6.6	22.5±6.1
c-PPAC Amount	28.4±12.7	27.5±11.1	29.2±14.4
c-PPAC Difficulty	50.4±12.8	53.8±11.5	47.6±13.4
c-PPAC Total	44.8±12.8	47.1±13.4	42.8±12.1
Steps per day	2551 (1463–3812)	2809 (1455–5398)	2292 (1471–3450)

Data are presented as mean±sd, n (%) or median (interquartile range). LVRS: lung volume reduction surgery; BLVR: bronchoscopic lung volume reduction; LTOT: long-term oxygen therapy; BMI: body mass index; FEV_1_: forced expiratory volume in 1 s; MRC: Medical Research Council; ISWT: incremental shuttle walk test; FVC: forced vital capacity; TLC: total lung capacity; RV: residual volume; FRC: functional residual capacity; *T*_LCO_: transfer factor of the lung for carbon monoxide; *K*_CO_: transfer coefficient of the lung for carbon monoxide; FFMI: fat-free mass index; CAT: COPD Assessment Test; c-PPAC: clinic visit PROactive Physical Activity in COPD. ^#^: self-reported in preceding year.

80 participants received treatment (34 LVRS/46 BLVR). Six randomised to the LVRS group and one randomised to the BLVR group decided against having the procedure post-randomisation and therefore exited the trial prior to treatment. One trial participant randomised to the LVRS group died before surgery was performed. These participants were not included in the intention-to-treat analysis. Follow-up of patients was interrupted due to the coronavirus disease 2019 (COVID-19) pandemic. Some in-person research visits were missed as they were not possible or considered unsafe in this vulnerable patient group. Where able, outcomes were collected over the phone. The COVID-19 pandemic also meant that access to some trial data was delayed, because research staff had been redeployed. Survival data at 12 months were available for all participants. Outcome data were available for 71 participants at 3 months (32 LVRS/39 BLVR) and 63 participants at 12 months (26 LVRS/37 BLVR). Complete primary end-point data (all i-BODE items at 12 months) were available for 49 participants (21 LVRS/28 BLVR) (figure 1) and in each of the composites as follows: BMI 22 LVRS/35 BLVR, FEV_1_ % pred 24 LVRS/33 BLVR, MRC dyspnoea score 26 LVRS/36 BLVR and ISWT 22 LVRS/32 BLVR.

**FIGURE 2:**
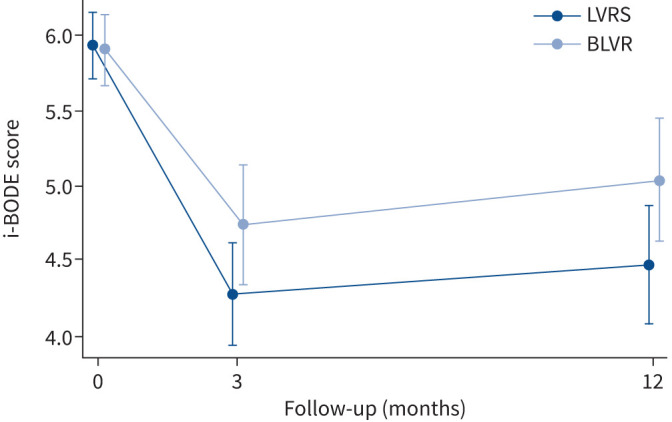
Effect of lung volume reduction interventions on i-BODE score. Data are presented as mean±sd for baseline, 3 months and 12 months post-procedure, and are based on all available data in the intention-to-treat population. LVRS: lung volume reduction surgery; BLVR: bronchoscopic lung volume reduction. Between-group difference at 12 months: p=0.54.

**FIGURE 3:**
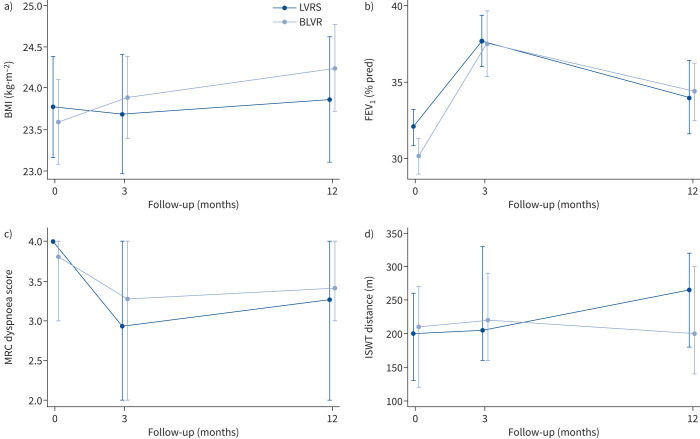
Effect of lung volume reduction procedures on i-BODE score component measures: a) body mass index (BMI), b) forced expiratory volume in 1 s (FEV_1_), c) Medical Research Council (MRC) dyspnoea score and d) incremental shuttle walk test (ISWT) distance. Data are presented as a, b) mean±sd or c, d) median (interquartile range) for baseline, 3 months and 12 months post-procedure and are based on all available data in the intention-to-treat population. LVRS: lung volume reduction surgery; BLVR: bronchoscopic lung volume reduction. Between-group difference at 12 months: a) p=0.16, b) p=0.11, c) p=0.19 and d) p=0.09.

**FIGURE 4:**
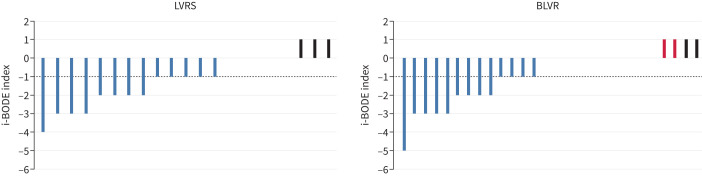
Responders based on minimal clinically important difference (MCID) for the i-BODE index (complete case analysis). Each bar represents an individual subject. Blue bars represent subjects that met or exceeded the MCID for the i-BODE index (−1 point). Black bars represent subjects who did not meet the MCID. Dotted line represents the MCID. Red bars represent those patients that either crossed over or had valves removed, where data was collected. LVRS: lung volume reduction surgery; BLVR: bronchoscopic lung volume reduction.

**FIGURE 5:**
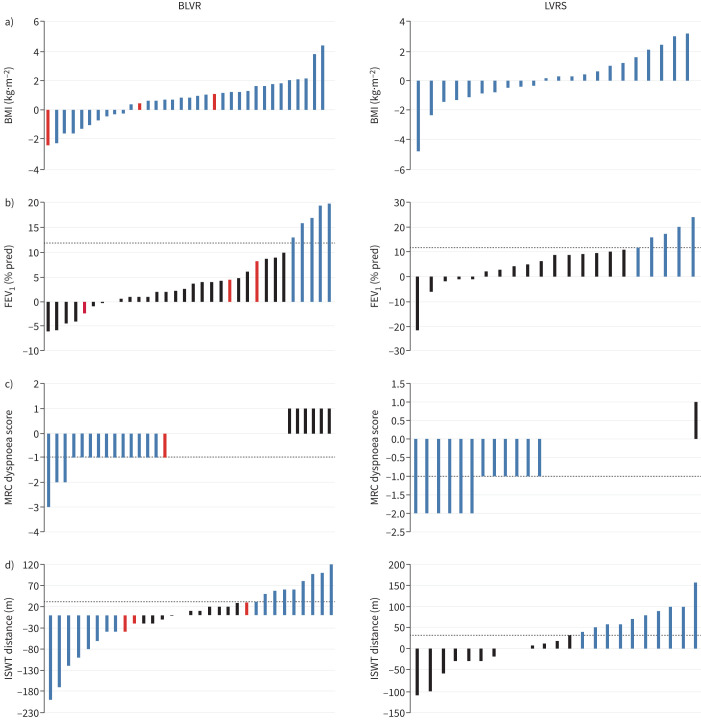
Responders based on minimal clinically important difference (MCID): i-BODE index components (complete case analysis). Each bar represents an individual subject. Blue bars represent subjects that met or exceeded the MCID for the specific outcome: a) body mass index (BMI) (no established MCID), b) forced expiratory volume in 1 s (FEV_1_) % pred (12%), c) Medical Research Council (MRC) dyspnoea score (−1 point) and d) incremental shuttle walk test (ISWT) distance (35 m). Black bars represent subjects who did not meet the MCID. Dotted line represents the MCID. Red bars represent those patients that either crossed over or had valves removed, where data was collected. BLVR: bronchoscopic lung volume reduction; LVRS: lung volume reduction surgery.

**FIGURE 6:**
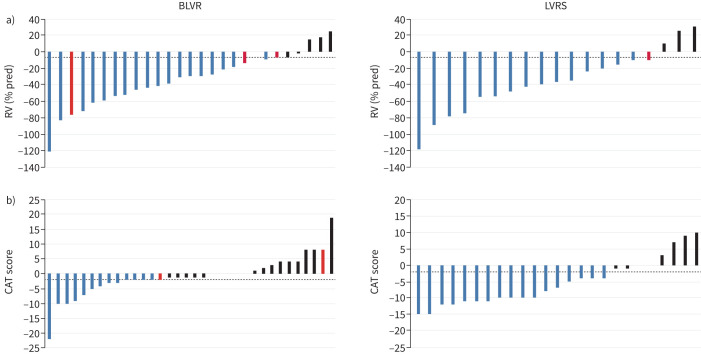
Responders based on minimal clinically important difference (MCID): important secondary outcomes (complete case analysis). Each bar represents an individual subject. Blue bars represent subjects that met or exceeded the MCID for a) residual volume (RV) % pred (−6.1%) and b) COPD Assessment Test (CAT) score (−2 points). Black bars represent subjects who did not meet the MCID. Dotted line represents the MCID. Red bars represent those patients that either crossed over or had valves removed, where data was collected. BLVR: bronchoscopic lung volume reduction; LVRS: lung volume reduction surgery.

At 12 months post-procedure both intervention groups showed an improvement in i-BODE score (LVRS −1.10±1.44 *versus* BLVR −0.82±1.61) with no significant difference between the two groups (treatment effect −0.27, 95% CI −0.62–1.17; p=0.54) (figure 2). Likewise, there was no difference reported in each of the four individual component measures that make up the i-BODE index between the two groups (table 2 and figure 3). A *post hoc* analysis showed that responder rates (using a fall of 1 point in the i-BODE index, as the score only allows whole number changes) at 12 months post-procedure were comparable between the two groups (48.8% in the LVRS group and 46.8% in the BLVR group (χ^2^=0.34, p=0.85)). Responder rates for components of the i-BODE index and for secondary outcomes are presented in the supplementary material and figures 4–6.

**TABLE 2:** Primary and secondary outcomes: change from baseline to 12 months follow-up

	**Change from baseline**		**Treatment effect (95% CI)**	**p-value**
	**LVRS**	**BLVR**		
**i-BODE score**	21: −1.10±1.44	28: −0.82±1.61	0.27 (−0.62–1.17)	0.54
**BMI (kg·m^−2^)**	22: 0.10±1.83	35: 0.74±1.57	0.64 (−0.27–1.56)	0.16
**FEV_1_ (% pred)**	24: 1.1±9.1	33: 4.5±6.8	3.4 (−0.8–7.6)	0.11
**MRC dyspnoea score**	26: −0.65±0.89	36: −0.33±0.97	−0.32 (−0.80–0.16)	0.19
**ISWT distance (m)**	22: 27.9±60.7	32: −4.8±73.8	−32.7 (−71.0–5.5)	0.09
**RV (% pred)**	19: −36.1 (−54.6– −10)	27: −30.1 (−53.7– −9)	2.7 (−25.4–19.1)	0.81
**CAT score**	25: −7 (−11– −1)	34: −1 (−3–3)	−6 (−9– −2)	0.005
**FFMI (kg·m^−2^)**	19: −0.79 (−3.67–1.44)	28: 0.46 (−1.84–1.89)	0.98 (−1.25–3.20)	0.39
**c-PPAC Amount**	18.0±19.7	15.3±14.5	−2.7 (−24.6–19.2)	0.79
**c-PPAC Difficulty**	17.2±14.4	12.0±17.9	−5.2 (−17.1–6.7)	0.38
**c-PPAC Total**	18.3±17.3	16.1±16.9	−2.2 (−15.8–11.4)	0.74
**Steps per day**	−478.5 (−1166–1102)	543 (−226–1332)	−847.5 (−2857–8726)	0.31

Change from baseline data are presented as number of participants data collected for, followed by mean±sd or median (interquartile range) change from baseline to 12 months follow-up; treatment effects are for bronchoscopic lung volume reduction (BLVR) *versus* lung volume reduction surgery (LVRS). BMI: body mass index; FEV_1_: forced expiratory volume in 1 s; MRC: Medical Research Council; ISWT: incremental shuttle walk test; RV: residual volume; CAT: COPD Assessment Test; FFMI: fat-free mass index; c-PPAC: clinic visit PROactive Physical Activity in COPD.

Both the LVRS and BLVR groups showed improvements in all secondary outcomes: RV % pred (LVRS −36.1% (95% CI −54.6– −10%) *versus* BLVR −30.1% (95% CI −53.7– −9%); p=0.81), physical activity experience (total c-PPAC measuring amount and difficulty) (LVRS +18.3±17.3 *versus* BLVR +16.1±16.9) and health-related quality of life (CAT) (LVRS −7 (95% CI −11–1) *versus* BLVR −1 (95% CI −3–3)). The only statistically significant between-group change in secondary outcomes was change in CAT score, which favoured those in the LVRS group (treatment effect −6, 95% CI –9– −2; p=0.005) (table 2 and supplementary figures S2–S4).

There were no differences at baseline between those with and without complete data at 12 months (supplementary table S1). This supported the data missing at random assumption, allowing a sensitivity analysis using multiple imputation, to derive data on missing items needed to calculate the composite i-BODE score. This showed similar results (i-BODE: LVRS −0.74±1.62 *versus* BLVR −0.89±1.43; treatment effect −0.15, 95% CI −0.89–0.53; p=0.66), while the CAT score showed a smaller difference between the two treatment arms at 12 months (LVRS −3.60±7.30 *versus* BLVR −0.04±7.58; treatment effect 3.56, 95% CI 0.18–6.93) (supplementary tables S2–S5). Of the study participants in the BLVR group who had complete RV follow-up data and achieved the minimum clinically important difference (MCID) (−6.1%) at 3 months, four (14.8%) no longer showed this benefit at 12 months. In the LVRS group this occurred in only one (5.3%) patient (figure 6).

Median (IQR) length of stay for the initial procedure was 9 (16.5) days in the LVRS group and 3 (2) days in the BLVR group (p=0.006). There were two deaths during the 12-month follow-up period. One occurred in the BLVR arm 44 days after valve insertion due to complications related to the procedure and one in the LVRS arm at 5 months post-intervention due to a non-infective acute exacerbation of COPD which was not thought to be related. At 12 months follow-up there were 29 respiratory-related adverse events in 17 participants undergoing LVRS (50.0%) *versus* 35 in 18 participants receiving BLVR (39.1%) (p=0.262, Fisher's exact test).

The most common complication was subcutaneous emphysema (29.3%) in the LVRS group and pneumothorax (30.4%) in the BLVR group. Of those who had a pneumothorax, nine (81.8%) occurred while still an inpatient post-procedure, median (IQR) time to onset 2 (3) days and drain removed after median (IQR) 10 (12) days. The median (IQR) number of days with a chest drain post-LVRS was 8.0 (11.0) days. 26 (59.1%) BLVR patients achieved complete lobar atelectasis and a further 10 (21.7%) partial atelectasis. Seven (15.0%) BLVR recipients required at least one further bronchoscopy or procedure following initial intervention and four (8.7%) crossed over to LVRS, within the 12-month follow-up period. In the LVRS arm, two (4.9%) required a further bronchoscopy or procedure and one (2.4%) crossed over to BLVR. Safety outcomes are presented in table 3. Further procedure-related details can be found in supplementary appendix 3.

**TABLE 3:** Safety outcomes

	**LVRS (n=34)**				**BLVR (n=46)**			
	**Subjects**		**Events**		**Subjects**		**Events**	
	**<30 days**	**1–12 months**	**<30 days**	**1–12 months**	**<30 days**	**1–12 months**	**<30 days**	**1–12 months**
**Any haemoptysis**	1 (2.9)	0	1	0	2 (4.3)	2 (4.3)	2	2
**Massive haemoptysis**	0	0	0	0	0	0	0	0
**Mortality**	0	1 (2.9)	0	1	1 (2.2)	0	1	0
**AE-COPD requiring hospitalisation**	2 (5.9)	1 (2.9)	2	1	2 (4.3)	7 (15.2)	2	7
**AE-COPD requiring NIV**	0	0	0	0	1 (2.2)	0	1	0
**AE-COPD requiring ICU** **stay**	0	0	0	0	0	0	0	0
**AE-COPD treated at home**	8 (23.5)	3 (8.8)	10	3	9 (19.6)	6 (13.0)	19	6
**Pneumonia**	0	1 (2.9)	0	1	2 (4.3)	1 (2.2)	2	2
**Pneumothorax**					14 (30.4)	1 (2.2)	14	1
**Post-surgical air leak**	4 (11)	0	5	0				
**Respiratory failure**	1 (2.9)	0	1	0	0	0	0	0
**Subcutaneous emphysema**	12 (35.3)	0	12	0	1 (2.2)	0	1	0
**Valve migration**					2 (4.3)	0	2	0
**Valve removal**					2 (4.3)	1 (2.2)	3	1
**Other repeat procedure**	2 (4.3)	0	2	0	5 (10.9)	0	5	0
**Prolonged stay post-procedure**	11 (32.4)				13 (28.3)			

Data are presented as n (%) or n. LVRS: lung volume reduction surgery; BLVR: bronchoscopic lung volume reduction; AE: acute exacerbation; NIV: non-invasive ventilation; ICU: intensive care unit. Serious adverse events were events leading to death, hospitalisation or prolongation of existing hospitalisation, persistent or significant disability/incapacity, or to serious deterioration in health that resulted in a life-threatening illness or injury, a permanent impairment of a body structure or body function. Prolonged length of stay defined as >10 days in LVRS and >4 days in BLVR.

## Discussion

The CELEB trial is the first randomised controlled trial to compare the effects of LVRS with BLVR. We found that surgery was not substantially superior to bronchoscopic treatment in patients with intact fissures and that both were similarly safe. Both approaches produced a clinically meaningful reduction in hyperinflation and similar improvements, assessed using either the i-BODE composite index or its individual components, were seen in both treatment arms at 1-year post-procedure. The initial length of hospital stay was longer following LVRS, but the BLVR group were more likely to have undergone a further intervention. There were also no significant differences found between the two groups in other secondary outcome measures (FFMI, physical activity experience and steps per day), with the exception of the CAT score which favoured LVRS at 12 months.

The use of a composite measure is considered a more meaningful way to evaluate prognosis and response to disease-modifying interventions than FEV_1_ alone [30] and indeed, due to the significantly heterogenous clinical phenotypes of COPD, combinations of several indices have better prognostic capability than any one outcome in isolation [31]. The i-BODE index was selected as our primary outcome, as differences in the BODE score at 3 months following LVRS have previously been shown to be associated with long-term survival [23] and it has also been shown to improve following BLVR [15, 32]. However, we do acknowledge the difficulties associated with using a measure that transforms continuous outcomes into categories, specifically that the likelihood of an individual making a meaningful change is determined by how close to a threshold they were for a baseline measurement and therefore may not represent the actual magnitude of change an individual has made. Although the study hypothesis was based on a “substantial” benefit favouring surgery, defined as a 1.5-point mean difference between groups, a difference of 1 point has been shown to be a significant predictor of prognosis when assessing COPD interventions [33, 34]. A *post hoc* responder analysis also found no difference between the proportions in each trial arm achieving this level (1 point) of benefit.

The greater the reduction in lung volume following lung volume reduction intervention, the greater the improvement in other outcomes such as lung function, exercise capacity and quality of life [4]. In terms of intervention efficacy, both LVRS and BLVR produced similar improvements in RV % pred, with both exceeding the MCID, defined as a 6.1% fall from baseline [35], by a clear margin.

The improvement in gas trapping observed in both study arms was accompanied by improvements in participants’ experience of physical activity assessed using the c-PPAC score of two to three times the established MCID of 6 points (c-PPAC Amount: LVRS 18.3 *versus* BLVR 15.3; c-PPAC Difficulty: LVRS 17.2 *versus* BLVR 12.0) [36]. The c-PPAC score, a combination of activity monitor data and subjective questionnaire, is considered a better method of measuring physical activity than a subjective or objective measure in isolation [27]. The magnitude of change seen in this outcome following lung volume reduction treatment would represent an important difference in a population who are greatly limited in everyday activities [37].

Safety outcomes were similar, with no statistical differences in adverse events between the two groups. There was no peri-operative (30-day) mortality in either group and a single death in each arm by 12 months. This rate is not more than would be expected without intervention in patients with this severity of disease and indeed a low mortality rate is expected given the survival benefit associated with effective lung volume reduction in people with COPD [38]. The most common complication in the BLVR arm (pneumothorax) occurred in 30.4% post-BLVR, which is consistent with other studies [39, 40], and occurred at a median 2 days post-procedure, which supports clinical practice of post-procedural inpatient observation, to allow this complication to be dealt with safely if it occurs [41]. Subcutaneous emphysema following LVRS, which can be distressing when severe [42], was the most common peri-operative complication (35.3%) in the surgical arm. Unfortunately, the severity of this was not documented so it is unclear whether this should be considered a significant adverse event or accepted as an anticipated complication.

Although LVRS required a longer initial hospital stay, it was associated with fewer subsequent procedures and more participants crossed over in the BLVR group (LVRS 1 (2.4%) *versus* BLVR 4 (8.5%)). In addition, the CAT score at 12 months favoured LVRS with a benefit exceeding the accepted MCID of 2 points, based on previous pulmonary rehabilitation and bronchodilator studies [43, 44]. This could reflect the need for fewer repeat procedures and the occurrence of numerically fewer exacerbations in the LVRS arm. As an isolated secondary end-point this must be interpreted with some caution, in particular as the use of a health status outcome in an unblinded trial can be subject to bias based on participants’ knowledge and expectations about the intervention. Furthermore, the sensitivity analysis using multiple imputations revealed a smaller difference between groups at 12 months that was no longer statistically significant. Of note, the CAT score was included in the mixed effects model used during data analysis.

We acknowledge a number of limitations and methodological issues with the present study. First, the findings relate to a very specific COPD phenotype, namely people who were considered to be suitable for both interventions, and cannot therefore be extrapolated to all people being considered for lung volume reduction. Some individuals have a non-anatomical pattern of emphysema where surgery may be more effective. Others may have comorbidities such as pulmonary hypertension that could preclude LVRS but where valve treatment could be considered. Second, although this first head-to-head study did not demonstrate that LVRS was substantially more effective than BLVR to an extent that would change existing clinical equipoise, that does not necessarily mean that they are equivalent and further larger trials will need to address this. Third, due in large part to the logistical difficulties conducting clinical visits during the COVID-19 pandemic, although we had complete data for survival, there were missing data for some end-points. However, the sensitivity analysis using a prespecified multiple imputation approach to missing data supports the headline findings of the study (supplementary table S5). We recognise that it may have been prudent to plan for a >10% dropout rate in our study design, given the known difficulties with participant crossover in interventional randomised controlled trials investigating surgical procedures, such as ours [45, 46]. These studies do, however, compare surgical intervention with no intervention and therefore must be considered in this context. Of note, the standard deviation for change in i-BODE score that we observed (1.44) was lower than that used in our sample size calculation. Re-calculating this *post hoc*, using that standard deviation, to exclude a 1.5-point difference between the two groups at a 90% power level and a significance level of 5% would require 20 patients in each treatment arm, suggesting that our trial was in fact adequately powered. Fourth, an unavoidable limitation in our trial was the lack of double blinding, as it was not possible to conceal treatment allocation from participants. We are aware that blinding of research staff collecting key outcomes would not completely address this bias and the use of a blinding questionnaire to address the magnitude of this confounding may have been appropriate [47]. A further potential source of bias may be considered in the lack of consistency in post-discharge care. For example, although all patients were required to undergo a course of pulmonary rehabilitation prior to enrolment on the trial, participation in post-intervention rehabilitation was not obligatory or documented. Given the well-documented effect pulmonary rehabilitation can have on many outcomes [48], including those investigated in this trial (FEV_1_, CAT and physical activity), the influence this may have had must be recognised. However, our study reflects differences in routine care pathways. A standardised pathway that necessitates post-intervention pulmonary rehabilitation may maximise patient outcomes from these interventions. Fifth, although the i-BODE index has not been formally validated within a lung volume reduction cohort, several studies have suggested that it may be of value in assessing patients post-LVRS [49, 50]. Finally, several participants withdrew from the trial following randomisation to LVRS during the interval between a surgery date being arranged. It is possible that more participants dropped out following randomisation to LVRS because they preferred to undergo what they believed to be a less invasive procedure, which may have introduced some bias into the findings. The topic of patient preference and satisfaction around undergoing an endoscopic *versus* a surgical procedure should be explored further. Our results are important for clinicians educating patients on these important procedures and in guiding informed decision making. Future research is called for to support these findings and a larger trial is already underway (SINCERE; ClinicalTrials.gov: NCT04537182). An economic evaluation of the CELEB trial data will increase understanding of the comparative value of the two approaches [24].

### Conclusions

The results of this study do not support the hypothesis that LVRS is a substantially superior treatment compared with BLVR, in terms of health outcomes achieved 1 year post-procedure. These broadly similar results at 12 months were obtained with a longer length of stay initially for LVRS but with less need for subsequent interventions than was the case with valve placement. The findings should help the lung volume reduction MDT to frame treatment options for patients and guide discussions around shared decision making, in individuals with severe COPD who are suitable for both LVRS and BLVR.

## Supplementary material

10.1183/13993003.02063-2022.Supp1 **Please note:** supplementary material is not edited by the Editorial Office, and is uploaded as it has been supplied by the author.
Supplementary material ERJ-02063-2022.Supplement

## Shareable PDF

10.1183/13993003.02063-2022.Shareable1 This one-page PDF can be shared freely online.
Shareable PDF ERJ-02063-2022.Shareable
