# Supplementary material for: Lung volume reduction surgery versus endobronchial valves: a randomised controlled trial
Source: Eur Respir J. 2023 Apr 27;61(4):2202063. doi: 10.1183/13993003.02063-2022 (PMC10133584; doi:10.1183/13993003.02063-2022)
Supplement: Supplementary file 1 [file ERJ-02063-2022.Supplement.pdf]

Online Data Supplement

Comparative Effectiveness of Lung Volume Reduction Surgery for Emphysema and Bronchoscopic lung volume reduction with valve placement: a randomised controlled trial.

Sara C Buttery[1,2], Winston Banya[1], Rocco Bilancia[3], Elizabeth Boyd[3], Julie Buckley[3] , Neil J Greening[4,5], Kay Housely[6] , Simon Jordan[2] , Samuel V Kemp[1], Alan J. B. Kirk[3], Lorna Latimer[4,5], Kelvin Lau[7] , Rod Lawson[6], Adam Lewis[8], John Moxham[9] , Sridhar Rathinam[5] , Michael C Steiner[4,5], Sara Tenconi[6] , David Waller[7], Pallav L Shah[1,2], Nicholas S Hopkinson,[1,2] , On behalf of the CELEB investigators*

Table of Contents for Online Data Supplement

Tables

Table S1: Comparing baseline characteristics of participants with and without primary outcome at 12 months follow up.

Table S2: 3 Months Follow up – Complete case Analysis.

Table S3: 12 Months Follow up – Complete case Analysis.

Table S4: Imputed Data Analysis for Primary and secondary outcome variables

Table S5: Primary and secondary outcomes: Change from Baseline to 12 months- complete case analysis and imputed data

Figures

Figure S1: Schematic outline of the trial design.

Figure S2: Mean change in secondary outcome measures at 3 months and 12 months post procedure in the LVRS group compared with the BLVR group.

Figure S3: Effect of lung volume reduction interventions on Residual Volume (%predicted)

Figure S4: Change in Physical activity outcomes at 3 months and 12 months post procedure in the LVRS group compared with the BLVR group.

Appendix

Appendix 1: Complete eligibility criteria

Appendix 2: Chartis, LVRS and BLVR procedure details

Appendix 3: Procedure related outcome details

Appendix 4: Individual participant detail: cross-overs and valve removal

Table S1: Comparing baseline characteristics of participants with and without primary outcome at 12 months follow up.

	Full i-BODE collected (n=49)	Full i-BODE not collected (n=39)	P value
Age (years)	64.4 (7.2)	64.8 (8.5)	0.82
Gender (M/F)	24/27	22/15	0.26
Exacerbations*,	2.3 (1.7)	2.8 (3.0)	0.34
A&E Attendances*,	0.4 (0.8)	0.4 (0.8)	0.31
Hospital admissions*,	0.4 (1.0)	0.4 (0.8)	0.40
Hospital days*,	1.7 (4.3)	1.9 (4.6)	0.42
i-BODE index score	5.8 (1.6)	6.1 (1.4)	0.29
BMI (kg/$m^{2)}$	23.6 (3.5)	23.4 (3.9)	0.60
$FEV_{1,}$ %predicted	31.2 (7.9)	30.9 (7.9)	0.88
MRC dyspnoea score	3.9 (0.7)	3.9 (0.8)	0.95
ISWT (m)	229.(120)	187 (90.0)	0.06
**Other Lung function parameters**			
FEV_1(l)	0.78 (0.20)	0.83 (0.23)	0.36
FVC, %predicted	89.1 (19.3)	82.1 (18.0)	0.09
TLC, %predicted	142.3 (13.4)	140.9 (15.7)	0.65
RV, %predicted	237.6 (36.8)	242.6 (41.8)	0.57
RV/TLC ratio	63.7 (5.9)	64.5 (7.1)	0.56
TLco, %predicted	35.6 (10.9)	36.1 (8.6)	0.80
Kco, %predicted	46.7 (14.1)	46.7 (13.7)	0.99
FFMI (kg.m^2)	30.8 (6.1)	31.0 (5.0)	0.93
CAT score	22.9 (6.6)	23.4 (6.1)	0.69

Data are presented as n (%) mean (SD) or median (IQR). i-BODE= composite health status measure made up of B=BMI; O=Obstruction (FEV_1%predicted); D= Dyspnoea (MRC score); E=Exercise capacity (ISWT).
BMI= body mass index. FEV_1= forced expiratory volume in 1 sec. MRC= Medical Research Council. ISWT= incremental shuttle walk test. FVC= forced vital capacity. TLC= total lung capacity. RV= residual volume. TLco= carbon monoxide transfer factor. Kco= carbon monoxide transfer coefficient. CAT= chronic obstructive pulmonary disease (COPD) assessment test score. FFMI= fat free mass index. * Self-reported in preceding year.

Table S2: 3 Months Follow up – based on all available data

Variable	All subjects (n=80)		LVRS (n=34)		BLVR (n=46)		p
	Missing	Statistic	Missing	Statistic	Missing	Statistic	
No. of Exacerbations	8	1 (0, 1)	3	0 (0, 1)	5	1 (0, 1)	0.15
A&E Attendances	8	0 (0, 0)	3	0 (0, 0)	5	0 (0, 0)	0.70
Hospital admissions	9	0 (0, 0)	4	0 (0, 0)	5	0 (0, 0.5)	0.19
Hospital days	27	0 (0, 0)	7	0 (0, 0)	20	0 (0, 0)	0.79
i-BODE score	17	4.5 (2.1)	6	4.3 (1.8)	11	4.7 (2.3)	0.39
BMI	10	23.8 (3.5)	7	23.7 (4.1)	3	23.9 (3.0)	0.82
MRC dyspnoea score	9	3.1 (1.0)	3	2.9 (1.0)	6	3.3 (1.1)	0.17
ISWT (M)	10	220 (160, 310)	3	205 (160, 330)	7	220 (160, 290)	0.81
FVC %	17	94.3 (20.5)	6	91.0 (18.9)	11	97.1 (21.7)	0.24
FEV_1/FVC	20	31.2 (8.3)	7	32.3 (9.2)	13	30.2 (7.5)	0.32
TLC (L)	24	7.4 (1.5)	10	7.2 (1.5)	14	7.6 (1.5)	0.31
TLC %	24	127.9 (13.9)	10	125.5 (14.5)	14	129.8 (13.2)	0.25
RV	24	4.3 (1.1)	10	4.2 (0.9)	14	4.3 (1.3)	0.63
RV %	24	188.8 (46.0)	10	185.3 (48.5)	14	191.6 (44.4)	0.61
RV%TLC	24	56.9 (8.8)	10	57.7 (8.3)	14	56.2 (9.2)	0.53
FRC	33	5.6 (1.3)	12	5.3 (1.2)	21	5.8 (1.4)	0.19
FRC %	33	174.5 (25.8)	12	172.1 (26.3)	21	176.7 (25.6)	0.54
TLCo	27	38.9 (12.9)	12	37.3 (11.1)	15	40.1 (14.1)	0.44
Kco	28	46.8 (12.8)	12	45.8 (12.8)	16	47.7 (12.9)	0.61
PaO_2	41	9.8 (1.4)	18	10.3 (1.6)	23	9.4 (1.2)	0.06
$PaCO_2$	41	4.82 (0.55)	18	4.78 (0.58)	23	4.85 (0.54)	0.69
CAT score	9	18.3 (8.4)	3	16.0 (8.2)	6	20.2 (8.2)	0.034
FFMI	14	30.6 (6.1)	5	31.0 (6.1)	9	30.2 (6.1)	0.63

Data are presented as mean(SD) or median (IQR). LVRS=lung volume reduction surgery. BLVR= bronchoscopic lung volume reduction surgery. BMI= body mass index. FEV_1= forced expiratory volume in 1 sec. MRC= Medical Research Council. ISWT= incremental shuttle walk test FVC= forced vital capacity. TLC= total lung capacity. RV= residual volume. FRC= functional residual capacity. TLco= carbon monoxide transfer factor. Kco= carbon monoxide transfer coefficient. PaO_2= arterial partial pressure of oxygen. $PaCO_2$=arterial partial pressure of carbon dioxide. CAT= chronic obstructive pulmonary disease (COPD) assessment test score. *Self-reported in preceding year.

Table S3: 12 Month Follow up –based on all available data

Variable	All subjects (n=80)		LVRS (n=34)		BLVR (n=46)		p
	Missing	Statistic	Missing	Statistic	Missing	Statistic	
AECOPD*	16	1 (0, 2)	7	1 (0, 2)	9	1 (0, 2)	0.97
A&E Attendance*	17	0 (0, 0)	7	0 (0, 0)	10	0 (0, 0)	0.44
Hospital admissions*	15	0 (0, 0)	6	0 (0, 0)	9	0 (0, 0)	0.92
Hospital days*	16	0 (0, 0)	6	0 (0, 0)	10	9 (0, 0)	0.43
iBODE score	31	4.8 (2.0)	13	4.5 (1.8)	17	5.0 (2.2)	0.38
BMI (kg/m^2)	21	24.1 (3.3)	11	23.9 (3.6)	10	24.2 (3.1)	0.68
FEV1 %	22	34.2 (11.0)	10	34.0 (11.7)	12	34.4 (10.7)	0.91
MRC dyspnoea score	17	3.35 (0.98)	8	3.27 (0.96)	9	3.42 (1.00)	0.61
ISWT (m)	25	235 (160, 310)	12	265 (180, 320)	13	210 (150, 310)	0.57
FVC (l)	22	2.96 (0.99)	10	2.84 (0.93)	12	3.04 (1.01)	0.44
FVC %	22	92.3 (28.0)	10	90.0 (30.5)	12	94.0 (26.4)	0.61
FEV_1/FVC	22	30.0 (6.5)	10	31.4 (6.3)	12	29.0 (6.5)	0.17
TLC (l)	32	7.5 (1.5)	14	7.4 (1.7)	18	7.6 (1.4)	0.63
TLC %	33	133.4 (16.3)	15	132.1 (16.7)	18	134.2 (16.2)	0.55
RV (l)	32	4.48 (1.15)	14	4.48 (1.39)	18	4.49 (0.96)	0.98
RV %	33	201.4(43.9)	15	199.6(48.4)	18	202.7(41.5)	0.81
RV%TLC	32	59.6 (9.6)	14	60.5 (11.2)	18	59.0 (8.3)	0.59
FRC (l)	36	5.7 (1.3)	15	5.6 (1.5)	21	5.8 (1.2)	0.57
FRC %	37	182.1(28.9)	16	181.2(32.6)	21	182.8(26.6)	0.86
TLco	37	38.4 (10.1)	18	37.8 (12.0)	19	37.3 (11.5)	0.77
Kco	36	49.0 (14.8)	18	48.9 (14.7)	18	49.1 (15.2)	0.97
PaO_2	44	9.7 (1.4)	23	10.3 (1.5)	21	9.4 (1.4)	0.08
$PaCO_2$	44	4.96 (0.56)	23	4.78 (0.61)	21	5.05 (0.53)	0.19
CAT	20	20.2 (8.1)	9	18.3 (8.0)	11	21.7 (7.9)	0.10
FFMI	29	30.5 (6.3)	13	28.9 (5.5)	16	31.7 (6.6)	0.12

Data are presented as mean(SD) or median (IQR). LVRS=lung volume reduction surgery. BLVR= bronchoscopic lung volume reduction surgery. BMI= body mass index. FEV_1= forced expiratory volume in 1 sec. MRC= Medical Research Council. ISWT= incremental shuttle walk test FVC= forced vital capacity. TLC= total lung capacity. RV= residual volume. FRC= functional residual capacity. TLco= carbon monoxide transfer factor. Kco= carbon monoxide transfer coefficient. PaO_2= arterial partial pressure of oxygen. $PaCO_2$=arterial partial pressure of carbon dioxide. CAT= chronic obstructive pulmonary disease (COPD) assessment test score. * Self-reported in preceding year.

Table S4: Imputed Data Analysis for Primary and secondary outcome variables

**Baseline**	**LVRS (n=41)**	**BLVR (n=47)**	**P**
i-BODE index score	5.9 (1.4)	5.9 (1.4)	0.92
BMI (kg/m^2)	23.6 (4.0)	23.6 (3.5)	0.83
FEV_1%predicted	32.0 (7.7)	30.2 (8.0)	0.27
MRC dyspnoea score	4 (4,4)	4 (3,4)	0.32
ISWT (m)	200 (130, 260)	210 (120, 270)	0.79
FEV1	0.81 (0.21)	0.80 (0.22)	0.82
RV % predicted	236.7 (38.8)	242.4 (38.7)	0.50
CAT	23.9 (6.6)	22.5 (6.1)	0.31
FFMI	31.0 (6.0)	30.8 (5.2)	0.89
**3 Months**			
**Variable**	**LVRS (n=34)**	**BLVR (n=46)**	**P**
i-BODE index score	4.4 (2.1)	4.9 (2.3)	0.38
BMI (kg/m^2)	23.4 (4.1)	24.1 (3.6)	0.45
FEV_1%predicted	37.7 (9.4)	37.1 (12.3)	0.82
MRC dyspnoea score	3 (2,4)	4 (2,4)	0.12
ISWT (m)	190 (11, 332)	220 (160, 290)	0.85
RV % predicted	190.7 (44.0)	195.8 (49.8)	0.63
CAT score	16 (8, 23)	21 (14, 27)	0.028
FFMI (kg/m^2)	31.0 (5.8)	31.2 (6.4)	0.89
**12 Months**			
**Variable**	**LVRS (n=34)**	**BLVR (n=46)**	**P**
i-BODE score	5.1 (21.)	5.0 (21.)	0.94
BMI (kg/m^2)	23.9 (4.0)	24.1 (3.4)	0.82
FEV_1%predicted	33.7 (10.8)	33.1 (11.6)	0.81
MRC dyspnoea score	3.5 (3,4)	3 (3,4)	0.80
ISWT (m)	212 (165, 310)	208 (157, 296)	0.76
RV % predicted	195.6 (62.6)	106.3 (46.2)	0.38
CAT score	20.5 (9.1)	22.4 (8.4)	0.36
FFMI (kg/m^2)	30.1 (6.3)	31.4 (6.5)	0.38

Data are presented as mean(SD) or median (IQR). LVRS=lung volume reduction surgery. BLVR= bronchoscopic lung volume reduction surgery. BMI= body mass index. FEV_1= forced expiratory volume in 1 sec. MRC= Medical Research Council. ISWT= incremental shuttle walk test FVC= forced vital capacity. TLC= total lung capacity. RV= residual volume. FRC= functional residual capacity. TLco= carbon monoxide transfer factor. Kco= carbon monoxide transfer coefficient. PaO_2= arterial partial pressure of oxygen. $PaCO_2$=arterial partial pressure of carbon dioxide. CAT= chronic obstructive pulmonary disease (COPD) assessment test score.
* Self-reported in precluding year.

Table S5: Primary and secondary outcomes: Change from Baseline to 12 months- complete case analysis and imputed data

Complete-Case Analysis

	**Difference: 12 months - Baseline**		**Treatment Effect (95% CI)**	**p**
	**LVRS**	**BLVR**		
i-BODE index score	21: -1.10 (1.44)	28: -0.82 (1.61)	0.27 (-0.62 to 1.17)	0.54
BMI (kg/m^2)	22: 0.10 (1.83)	35: 0.74 (1.57)	0.64 (-0.27 to 1.56)	0.16
FEV1 %predicted	24: 1.1 (9.1)	33: 4.5 (6.8)	3.4 (-0.8 to 7.6)	0.11
MRC dyspnoea score	26: -0.65 (0.89)	36: -0.33 (0.97)	-0.32 (-0.80 to 0.16)	0.19
ISWT (m)	22: 27.9 (60.7)	32: -4.8 (73.8)	-32.7 (-71.0 to 5.5)	0.09
FFMI (kg/m^2)	19: -0.79 (-3.67, 1.44)	28: 0.46 (-1.84, 1.89)	0.98 (-1.25 to 3.20)	0.39
RV % predicted	19: -36.1 (-54.6, -10)	26: -30.1 (-53.7, -9)	-2.7 (-25.4 to 19.1)	0.81
CAT score	25: -7 (-11, -1)	34: -1 (-3, 3)	6 (2 to 9)	0.005

Imputed data

**Variable**	**Difference: 12 months - Baseline**		**Treatment effect (95%CI)**	**P**
	**LVRS**	**BLVR**		
i-BODE score index	-0.74 (1.62)	-0.89 (1.43)	-0.15 (-0.84 to 0.53)	0.66
BMI (kg/m^2)	0.07 (1.74)	0.64 (1.48)	0.57 (-0.15 to 1.29)	0.12
MRC dysponea score	-0.50 (1.02)	-0.40 (0.89)	0.1 (-0.33 to 0.53)	0.64
FEV1 %PRED	1.3 (8.5)	2.8 (8.0)	1.5 (-2.3 to 5.2)	0.44
ISWT (m)	21.6 (67.1)	9.2 (74.7)	-12.4 (-30.5 to 1.5)	0.45
FFMI (kg/m^2)	-0.34 (-2.49, 1.43)	0.51 (-2.95, 3.17)	0.81 (-0.95 to 2.81)	0.38
RV % predicted	-32.47 (-55.66, -10)	-29.10 (-59, -9.67)	-0.38 (-17.60 to 17.96)	0.99
CAT score	-3.60 (7.30)	-0.04 (7.58)	3.56 (0.18 to 6.93)	0.04

Data are presented as mean(SD) or median (IQR). LVRS=lung volume reduction surgery. BLVR= bronchoscopic lung volume reduction surgery. BMI= body mass index. FEV_1= forced expiratory volume in 1 sec. MRC= Medical Research Council. ISWT= incremental shuttle walk test FVC= forced vital capacity. TLC= total lung capacity. RV= residual volume. FRC= functional residual capacity. TLco= carbon monoxide transfer factor. Kco= carbon monoxide transfer coefficient. PaO_2= arterial partial pressure of oxygen. $PaCO_2$=arterial partial pressure of carbon dioxide. CAT= chronic obstructive pulmonary disease (COPD) assessment test score. * Self-reported in preceding year.

Figure S1: Schematic outline of the trial design.

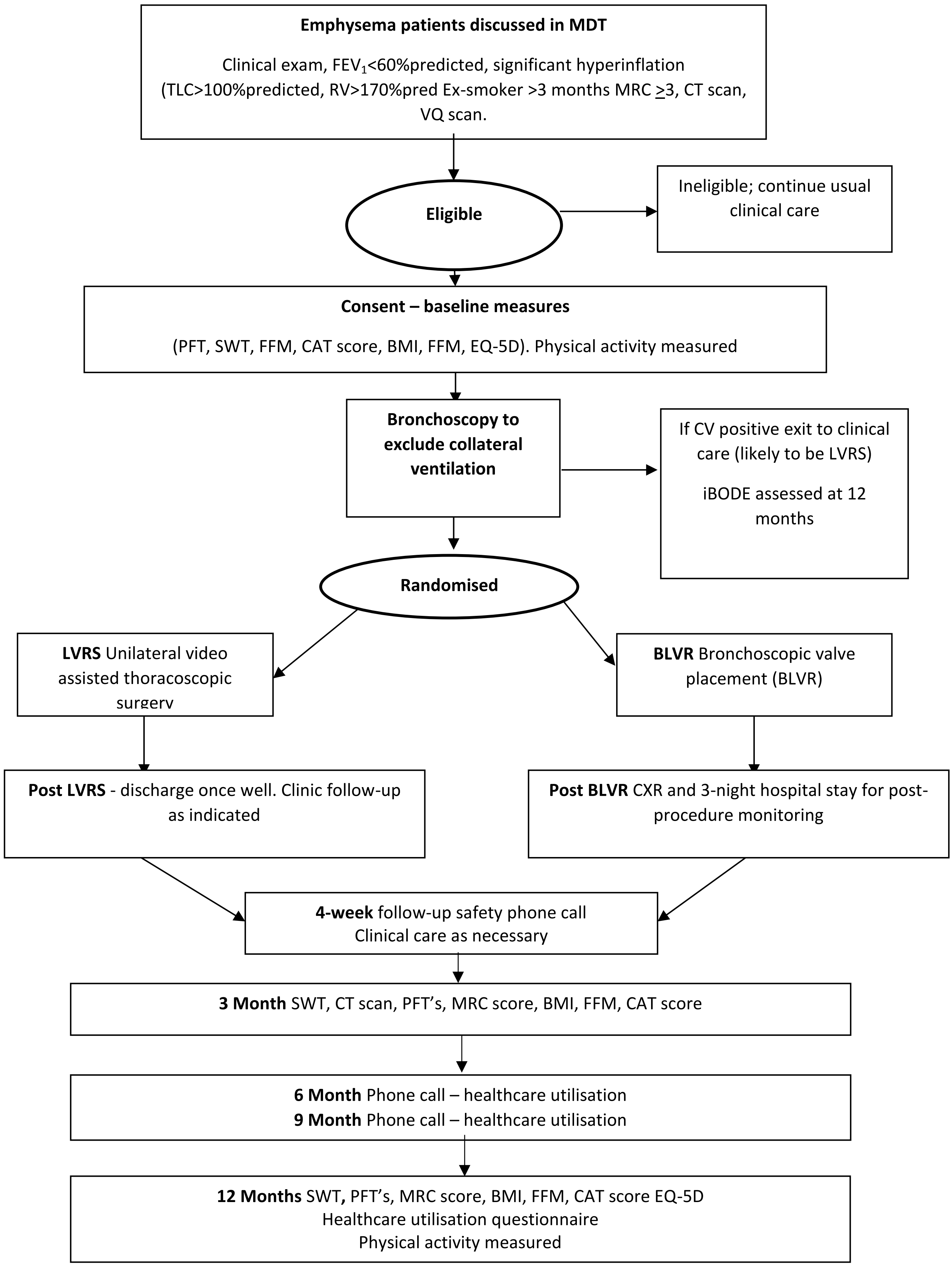

Legend to Figure S1: BLVR, bronchoscopic lung volume reduction; BMI, body mass index; CAT, COPD Assessment Test; CV, collateral ventilation; CXR, chest X-ray; FFM, fat-free mass; iBODE, a composite score including BMI, airflow obstruction, dyspnoea and exercise capacity (incremental shuttle walk test); LVRS, lung volume reduction surgery; MDT, multidisciplinary team; MRC, Medical Research Council; PFT, pulmonary function tests, RV, residual volume; SWT, shuttle walk test; TLC, total lung capacity; VQ lung ventilation/perfusion scan

Figure S2: Mean change in secondary outcome measures at 3 months and 12 months post procedure in the LVRS group compared with the BLVR group.

Figure S2a: RV% predicted

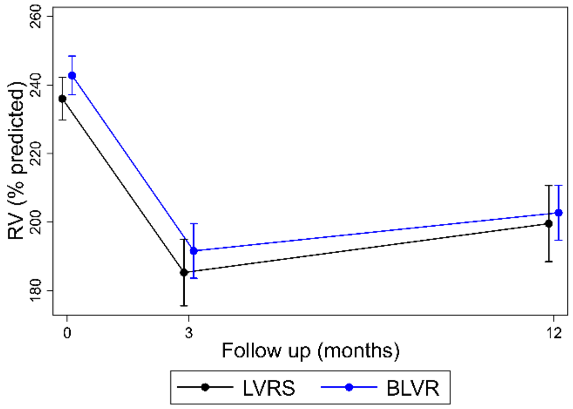

Figure S2b: CAT score

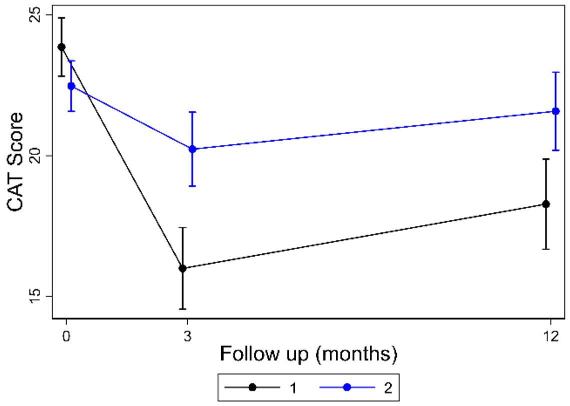

Figure S2c: FFMI

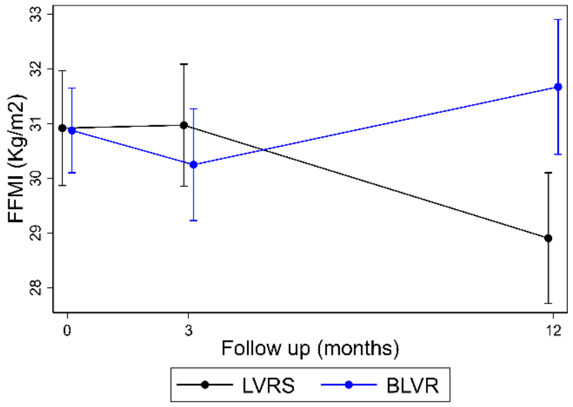

Figure S2d: Total C-PACC score

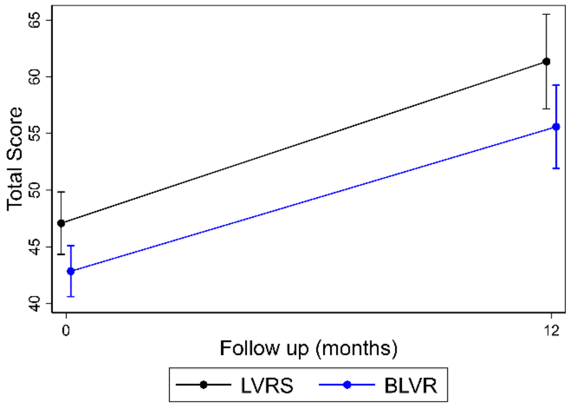

Legend to figure S2: Data presented are mean (SD) for baseline, 3 months and 12 months post procedure. LVRS: Lung volume reduction surgery; BLVR: Bronchoscopic lung volume reduction. RV: Residual Volume; CAT score: chronic obstructive pulmonary disease (COPD) assessment test score; FFMI; Fat free mass index; C-PACC score: Clinical visit-PROactive Physical Activity in COPD. Figure S2a p=0.81; Figure S2b p=0.005; Figure S2c p=0.39; Figure S2d p=0.74.

Figure S3: Effect of lung volume reduction interventions on Residual Volume (%predicted)

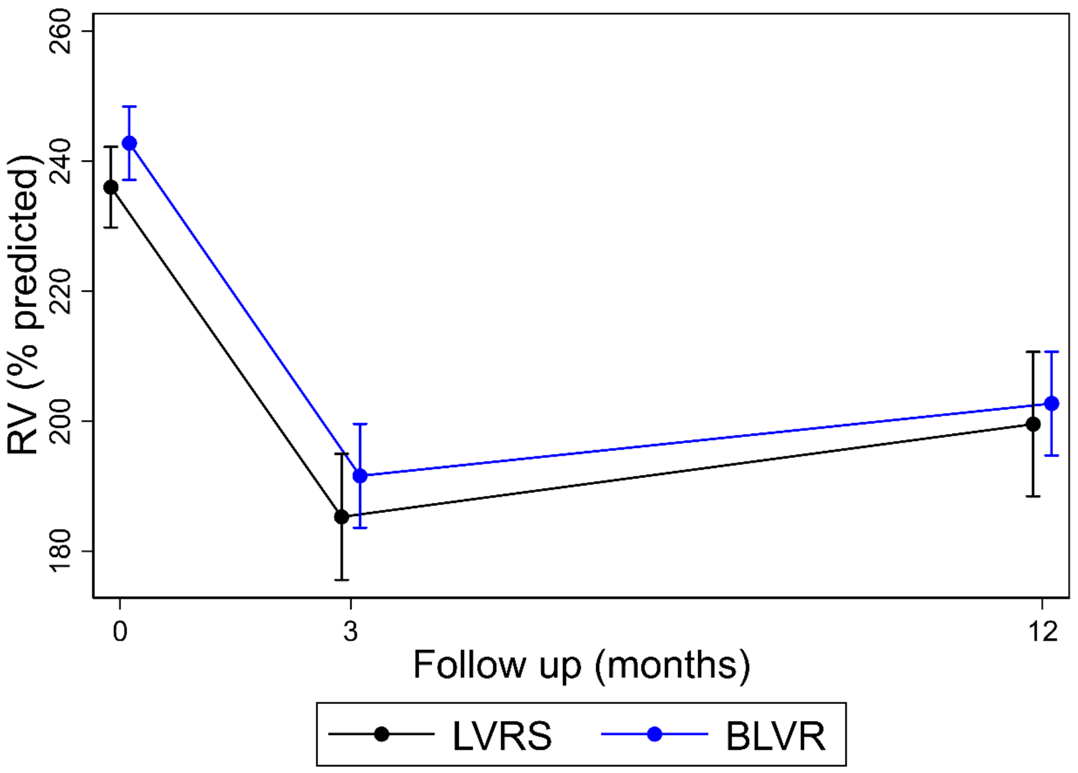

Legend to figure S3: Data presented are mean (SD) for baseline, 3 months and 12 months post procedure. Lung volume reduction surgery; BLVR: Bronchoscopic lung volume reduction. RV: Residual Volume. Between group difference at 12 months; p=0.81

Figure S4: Change in Physical activity outcomes at 3 months and 12 months post procedure in the LVRS group compared with the BLVR group.

Figure S3a: C-PPAC Amount score

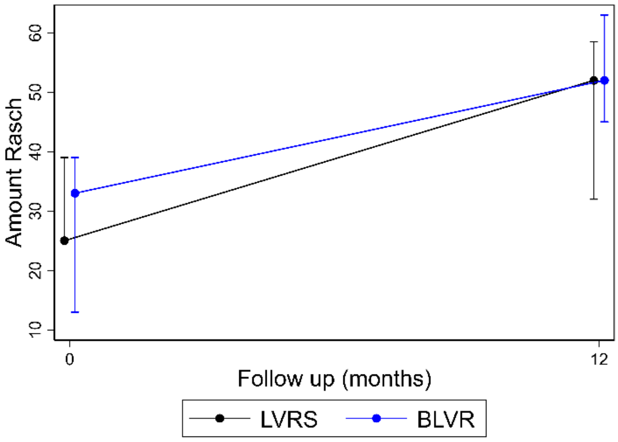

Figure S3b: C-PPAC Difficulty score

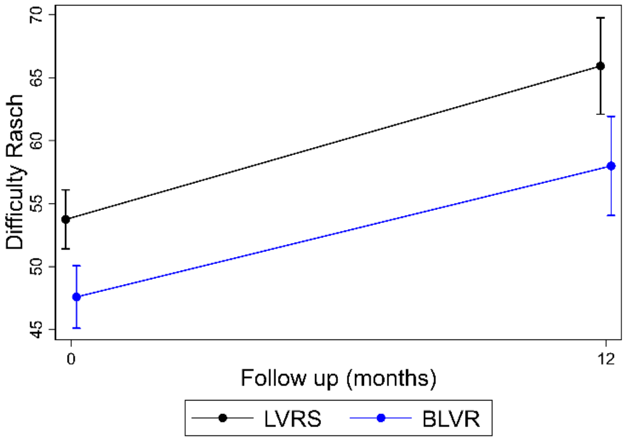

Figure S3d: C-PPAC Total score

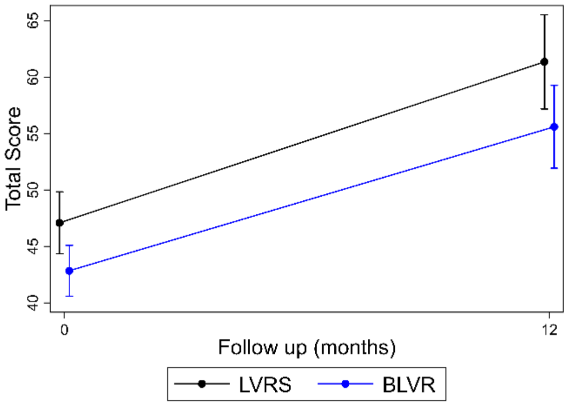

Figure S3c: Steps per day

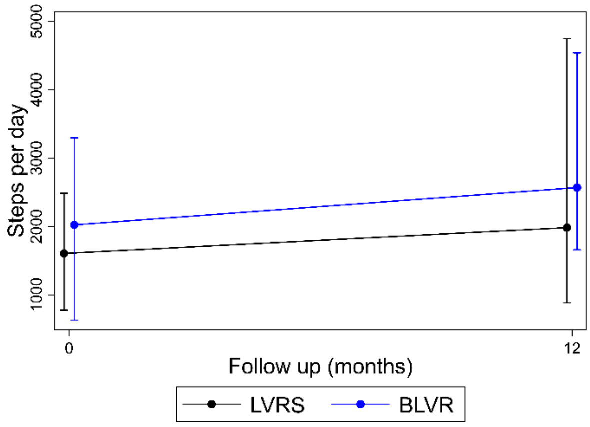

Legend to figure S3: Data presented are mean (SD) for baseline, 3 months and 12 months post procedure. LVRS: Lung volume reduction surgery; BLVR: Bronchoscopic lung volume reduction; compared with the BLVR group. C-PACC score: Clinical visit-PROactive Physical Activity in COPD. Figure S3a p=0.79; Figure S3b p=0.38; Figure S3c p=0.74; Figure S3d p=0.39

Figure S5: Responders based on Minimal Clinically Important Difference for the i-BODE index (complete case analysis)

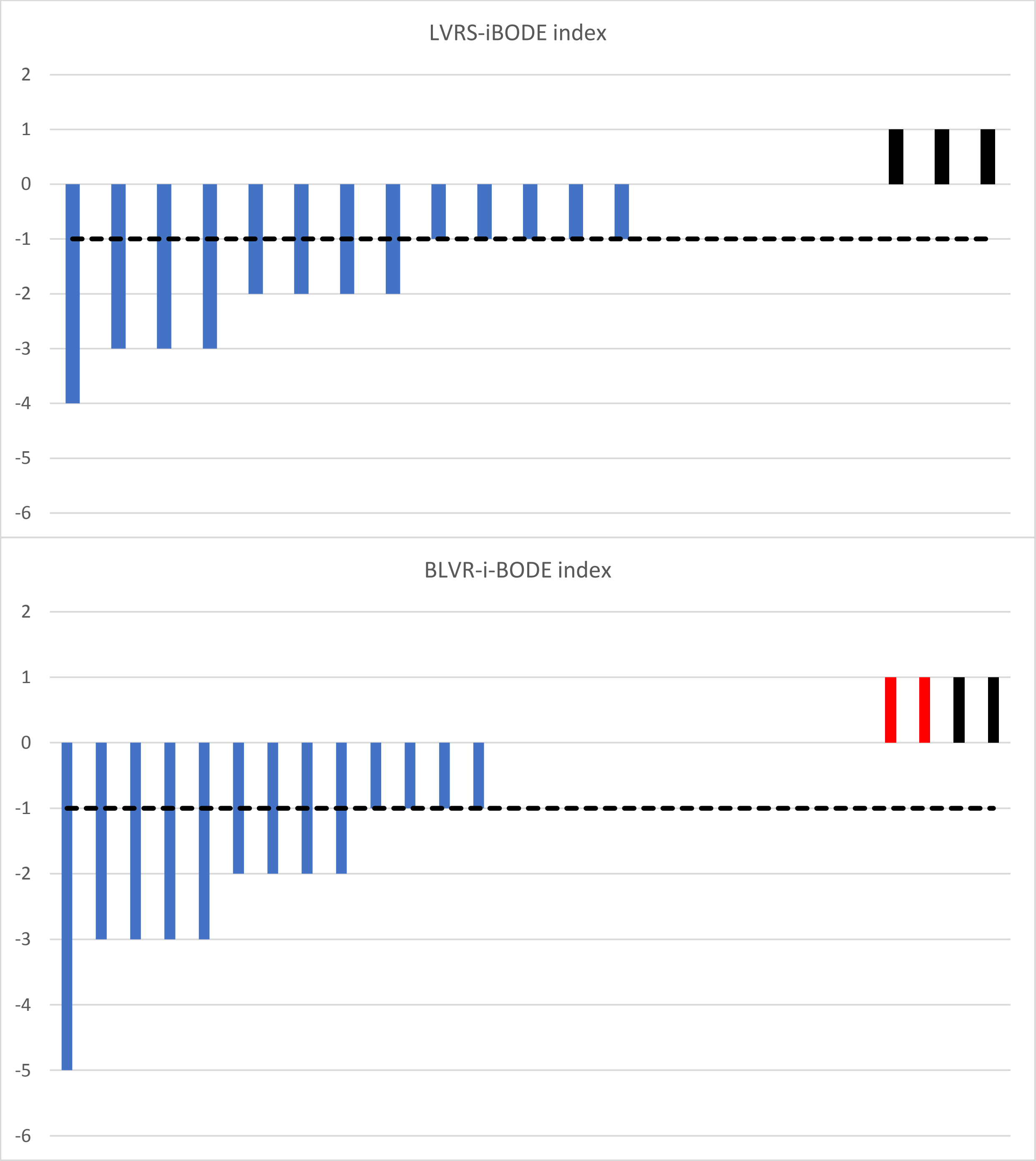

Legend to Figure S5: Each bar represents an individual subject. Blue bars represent subjects that met or exceeded the minimal clinical important difference (MCID) for the iBODE index: -1 points; Black bars represent subjects who did not meet the MCID. Dotted line represents the MCID. Red bars represent those patients that either crossed over or had valves removed, where data was collected.

Figure S6: Responders based on Minimal Clinically Important Difference- i-BODE index components (complete case analysis)

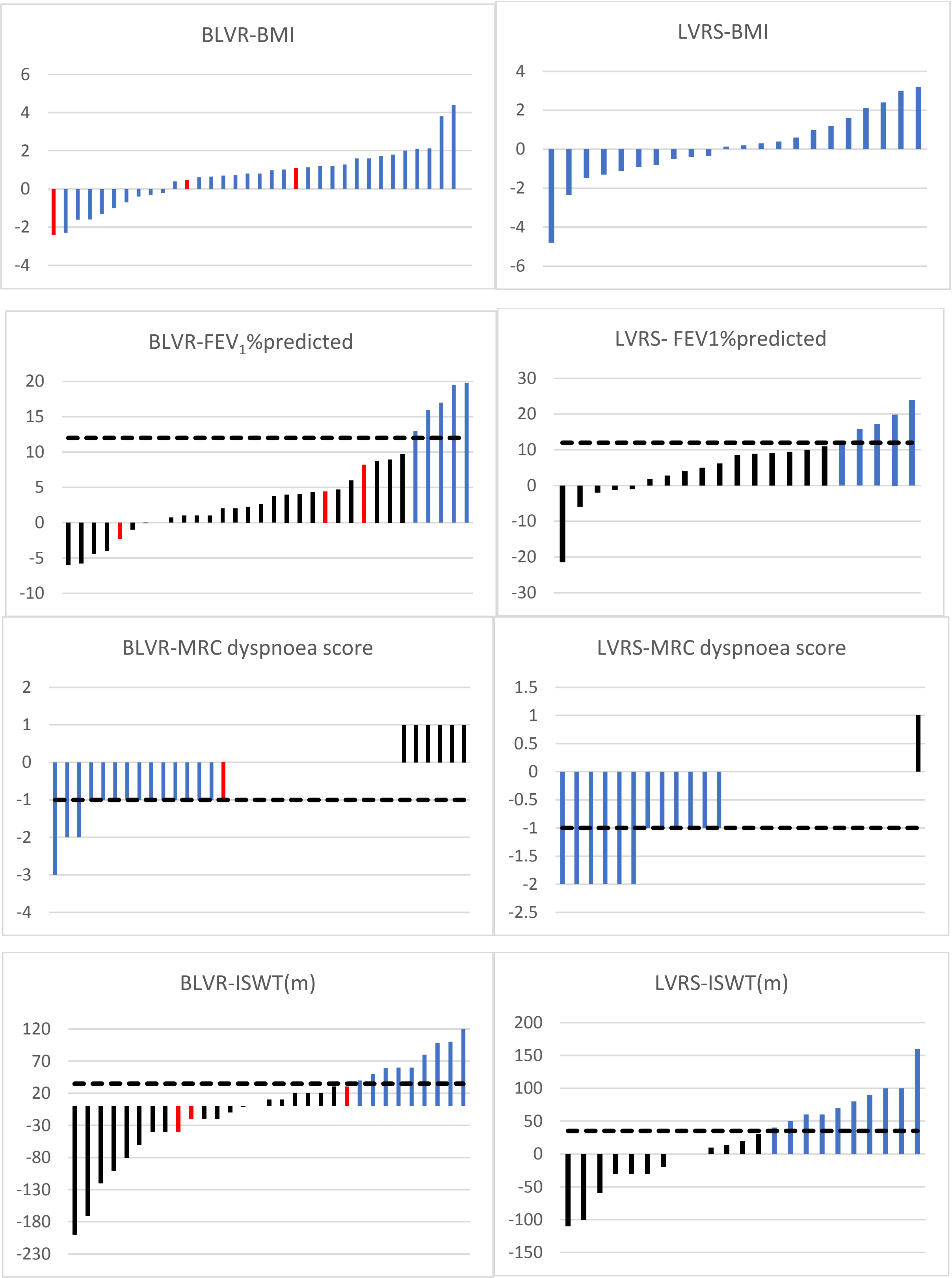

Legend to Figure S6: Each bar represents an individual subject. Blue bars represent subjects that met or exceeded the minimal clinical important difference (MCID) for the specific outcome; a) BMI: no established MID; b) FEV_1%predicted: 12%; c) MRC dyspnoea score: -1 points; d) ISWT: Black bars represent subjects who did not meet the MCID. Dotted line represents the MCID. Red bars represent those patients that either crossed over or had valves removed, where data was collected.

Figure S7: Responders based on Minimal Clinically Important Difference- Important secondary outcomes

(complete case analysis)

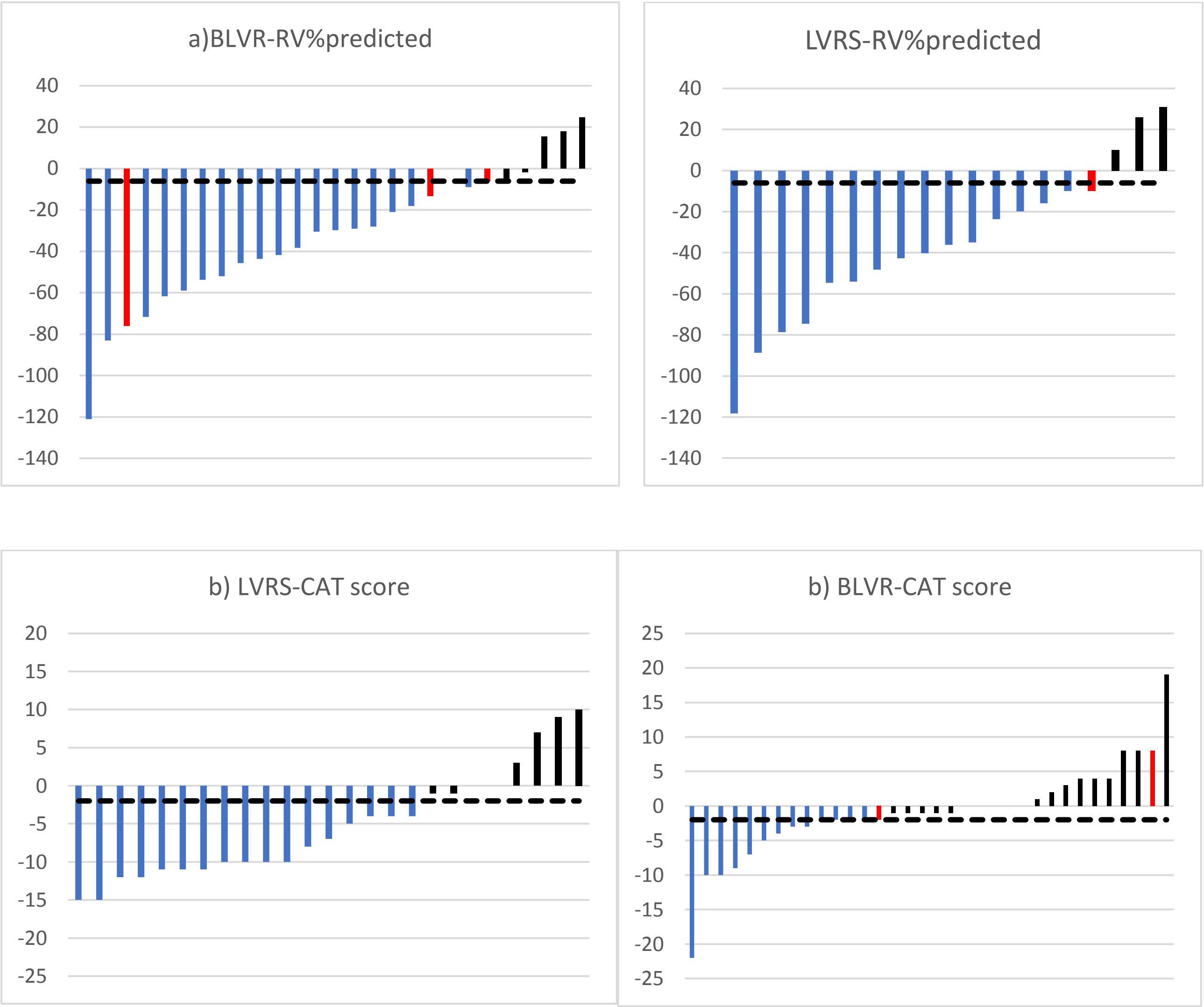

Legend to Figure S7: Each bar represents an individual subject. Blue bars represent subjects that met or exceeded the minimal clinical important difference (MCID) for a) RV%predicted (-6.1%) b) CAT score (-2 points) Black bars represent subjects who did not meet the MCID. Dotted line represents the MCID. Red bars represent those patients that either crossed over or had valves removed, where data was collected.

Appendix 1

CELEB trial eligibility criteria

<table>
<tr><td rowspan="2">Eligibility criteria:</td><td>Inclusion criteria:
▪ adults with COPD
▪ FEV_1 <60%
▪ TLC>100%
▪ RV>170%
▪ assessed at MDT to have intact interlobar fissures and heterogeneous emphysema and a candidate for LVRS or BLVR based on thoracic CT and lung perfusion, lung function and functional performance data.</td></tr>
<tr><td>Exclusion criteria:
▪ smoking within 3 months
▪ CT scan shows interlobar fissures are not intact
▪ major comorbidity limiting survival
▪ significant pulmonary fibrosis
▪ FEV_1 and TLco <20%
▪ PaO_2 < 7.0kPa
▪ $PaCO_2$ > 7kPa
▪ Collateral ventilation assessed by Chartis™ system.</td></tr>
</table>

Appendix 2

Chartis procedure

The Chartis™ Pulmonary Assessment System (PulmonX, Redwoood) consists of a single-patient-use catheter with a compliant balloon component at the distal tip, which upon inflation blocks the airway. Air can then flow out from the target compartment into the environment only through the Chartis catheter's central lumen. By connecting to a Chartis console, airway flow and pressure can be displayed. Airway resistance can be calculated and Collateral ventilation (CV) in isolated lung compartments can be measured (1)

Appendix 3

Treatment Details

A median of 4 valves (range 2-7) per subject were placed in the 46 BLVR subjects. Treatment distributions were as follows; 17 (37%) Left upper lobe, 9 (19.7%) left lower lobe, 11 (23.9%) right upper lobe, 4 (8.7%) right lower lobe. 4 (8.7%) right upper and middle lobe combined and 1 (2.2%) right middle and lower lobe combined. In the LVRS arm 19 participants received right sided LVRS, and 15 left sided LVRS. 30 participants received video-assisted thorascopic surgery (VATS) whilst 2 received robot-assisted thorascopic surgery (RATS) and 2 underwent a thoracotomy. All participants in the LVRS arm underwent unilateral procedures.

Appendix 4;

Individual participant details: Repeat procedures, cross-overs and valve removals

In the LVRS arm one participant went back to theatre for EBV insertion due to a prolonged air leak (cross-over) and one participant had a redo thoracotomy and wash out of haemothorax.

There were seven repeat procedures in the BLVR group requiring the participant to undergo a further bronchoscopy; 4 related to pneumothoraces with two requiring surgical chest drains and 2 undergoing blood pleurodesis. Two participants had valves removed and one participant had valves removed and re-placed before undergoing a LVRS (cross-over). Three further participants in the BLVR arm crossed over into the LVRS arm due to no symptomatic benefit.

References

1. Herth FJ, Eberhardt R, Gompelmann D, Ficker JH, Wagner M, Ek L, et al. Radiological and clinical outcomes of using Chartis™ to plan endobronchial valve treatment. European Respiratory Journal. 2013;41(2):302-8.
