# Supplementary material for: Lung volume reduction surgery versus endobronchial valves: a randomised controlled trial
Source: Eur Respir J. 2023 Apr 27;61(4):2202063. doi: 10.1183/13993003.02063-2022 (PMC10133584; doi:10.1183/13993003.02063-2022)
Supplement: Supplementary file 2 [file ERJ-02063-2022.Shareable.pdf]

EUROPEAN RESPIRATORY JOURNAL
ORIGINAL RESEARCH ARTICLE
S.C. BUTTERY ET AL.

SHAREABLE PDF

Lung volume reduction surgery *versus* endobronchial valves: a randomised controlled trial

Sara C. Buttery[1,2], Winston Banya[1], Rocco Bilancia[3], Elizabeth Boyd[3], Julie Buckley[3], Neil J. Greening[4,5], Kay Housley[6], Simon Jordan[2], Samuel V. Kemp[1], Alan J.B. Kirk[3], Lorna Latimer[4,5], Kelvin Lau[7], Rod Lawson[6], Adam Lewis[8], John Moxham[9], Sridhar Rathinam[5], Michael C. Steiner[4,5], Sara Tenconi[6], David Waller[7], Pallav L. Shah[1,2] and Nicholas S. Hopkinson[1,2] on behalf of the CELEB investigators

[1]National Heart and Lung Institute, Imperial College London, London, UK. [2]Royal Brompton and Harefield Hospitals, London, UK. [3]Department of Thoracic Surgery, West of Scotland Regional Heart and Lung Centre, Golden Jubilee National Hospital, Glasgow, UK. [4]Department of Respiratory Sciences, University of Leicester, Leicester, UK. [5]Institute for Lung Health, National Institute for Health Research Leicester Biomedical Research Centre – Respiratory, Glenfield Hospital, Leicester, UK. [6]Northern General Hospital, Sheffield, UK. [7]St Bartholomew's Hospital, London, UK. [8]Department of Health Sciences, Brunel University London, Uxbridge, UK. [9]King's College, London, UK.

Corresponding author: Nicholas S. Hopkinson (n.hopkinson@ic.ac.uk)

Check for updates

Shareable abstract (@ERSpublications)
In this first randomised study to compare lung volume reduction surgery and endobronchial valve placement in people who are suitable for both treatments, surgery did not produce substantially superior outcomes at 1 year post-procedure http://bit.ly/3D0DjoN

Cite this article as: Buttery SC, Banya W, Bilancia R, *et al*. Lung volume reduction surgery *versus* endobronchial valves: a randomised controlled trial. *Eur Respir J* 2023; 61: 2202063 [DOI: 10.1183/13993003.02063-2022].

This single-page version can be shared freely online.

Copyright ©The authors 2023.

This version is distributed under the terms of the Creative Commons Attribution Licence 4.0.

This article has an editorial commentary: https://doi.org/10.1183/13993003.00353-2023

Received: 26 Oct 2022
Accepted: 13 Jan 2023

Abstract

Background Lung volume reduction surgery (LVRS) and bronchoscopic lung volume reduction (BLVR) with endobronchial valves can improve outcomes in appropriately selected patients with emphysema. However, no direct comparison data exist to inform clinical decision making in people who appear suitable for both procedures. Our aim was to investigate whether LVRS produces superior health outcomes when compared with BLVR at 12 months.

Methods This multicentre, single-blind, parallel-group trial randomised patients from five UK hospitals, who were suitable for a targeted lung volume reduction procedure, to either LVRS or BLVR and compared outcomes at 1 year using the i-BODE score. This composite disease severity measure includes body mass index, airflow obstruction, dyspnoea and exercise capacity (incremental shuttle walk test). The researchers responsible for collecting outcomes were masked to treatment allocation. All outcomes were assessed in the intention-to-treat population.

Results 88 participants (48% female, mean±SD age 64.6±7.7 years, forced expiratory volume in 1 s percent predicted 31.0±7.9%) were recruited at five specialist centres across the UK and randomised to either LVRS (n=41) or BLVR (n=47). At 12 months follow-up, the complete i-BODE was available in 49 participants (21 LVRS/28 BLVR). Neither improvement in the i-BODE score (LVRS −1.10±1.44 *versus* BLVR −0.82±1.61; p=0.54) nor in its individual components differed between groups. Both treatments produced similar improvements in gas trapping (residual volume percent predicted: LVRS −36.1% (95% CI −54.6– −10%) *versus* BLVR −30.1% (95% CI −53.7– −9%); p=0.81). There was one death in each treatment arm.

Conclusion Our findings do not support the hypothesis that LVRS is a substantially superior treatment to BLVR in individuals who are suitable for both treatments.
